# Synergistic effect of benzo triazole with polyethoxylated sorbitan monooleate in inhibiting corrosion of rebars and chloride diffusion through mortars

**DOI:** 10.1038/s41598-024-65962-w

**Published:** 2024-07-02

**Authors:** Raja Rizwan Hussain, Abdulrahman Alhozaimy, Abdulaziz Al-Negheimish, D. D. N. Singh, Mshtaq Ahmed

**Affiliations:** 1 Civil Engineering Department, Center of Excellence for Concrete Research and Testing (CoE-CRT), College of Engineering, King Saud University, PO Box: 800, 11421 Riyadh, Saudi Arabia; 2 Corrosion and Surface Engineering CSIR, National Metallurgical Laboratory, Jamshedpur, India; 3 College of Engineering, King Saud University, Riyadh, Saudi Arabia

**Keywords:** Chemistry, Engineering, Materials science, Nanoscience and technology

## Abstract

It is found that mixture of 1,2,3 benzo triazole (BTAH) with polyethoxylated sorbitan monooleate, a non-ionic surface-active agent (NIS) effectively improves the properties of the cast concrete as well as significantly reduces the chloride induced corrosion of steel reinforced bars, when added in freshly prepared paste of mortar mixture. The addition of this mixture in the cast mortars is noted to reduce the water absorption in comparison to the control mortars cast using identical materials and under similar cast conditions. Electrochemical impedance spectroscopy and polarization studies of the rebars embedded in mortars and exposed in cement slurry have been performed to study the role of synergistic mixture on kinetics and mechanism of corrosion of rebars. The characterisation of corrosion products formed on the surface of rebars was carried out by X-ray diffraction, Scanning electron microscopy and EDX analysis. It is proposed that the synergistic boosting in protection is caused due to the shielding of NIS around anionic BTA^−^, thus minimizing their electrostatic repulsion. This facilitates the migration of additional ionic BTA towards the double layer which increases their concentration at the corroding interface leading to reduced susceptibility to corrosion.

## Introduction

Amongst different factors responsible for the deterioration of concrete structures poor mechanical properties, uniform and localized corrosion of embedded steel rebars caused due to water and moisture absorption and carbonation during service life play very crucial role in determining their longevity. A primary objective of civil engineers is to design and develop concrete structures with improved workability of fresh concrete, particularly in hot climatic regions, higher strength, and durability at minimal cost. Use of corrosion inhibitors in wet concrete mixture is very cost effective and simple method to keep the embedded rebars intact during service life of the cast structures^1–3^. In many instances the added corrosion inhibitors adversely affect other properties such as mechanical properties, setting time and porosity of concrete. Achieving all the above stated properties of concrete by a single additive is a big challenge and in majority of the cases multiple additions are needed to get the desired results. Such multiple additives not only escalate the cost of structures but in many cases also nullify or deteriorate the efficacy of the different components of the additives. In view of this it is prudent to look for some synergistic mixture that helps in improving all the desired properties namely workability, water absorption, strength, corrosion resistance of embedded rebars and overall durability of the cast structures.

Amongst various causes that deteriorate the concrete structures, two factors namely chloride diffusion in concrete and its carbonation bringing down the alkalinity are of prime concern^4^. Accumulation of chloride above a threshold concentration around the concrete-reinforcement bars causes localized attack leading to catastrophic failures of structures. To minimize this risk, it is important either to reduce the diffusion of chloride through the cast concrete or increase the threshold concentration of chloride for onset of corrosion on the surface of reinforcement. Mixing of pozzolanic materials in concrete at the time of its casting minimize the chloride diffusion in hard structures^5,6^. These pozzolanic materials densify the structure of concrete creating tortuous path for migration of surrounding chloride through it. Application of cathodic protection on whole network of reinforcement is another method to increase the threshold concentration of chloride to prevent its damaging effect^7,8^. Application of optimized cathodic potential or current makes the interface negatively charged which repels chloride anions away from the surface of reinforcement. This method of protection, however considerably escalates the cost of construction and needs regular care and maintenance. Another method to keep away chloride ions from the surface of steel reinforcement and increase its threshold concentration for attack on rebars is use of appropriate corrosion inhibitors. Many exhaustive reviews are available in literature on this topic^9,10^. Two types of inhibitors namely inorganic and organic chemicals are employed to control corrosion of reinforcement bars. Inorganic compounds such as nitrites, borates, nitrates etc. are used to mitigate the corrosion reactions. These compounds are anodic type of inhibitors and help in strengthening the passive film of the metal surface. Their performances however are concentration sensitive and cause acceleration of corrosion when used at lower concentrations. Organic inhibitors control the corrosion reactions by getting adsorbed on corroding interface through their active centres and inhibit anodic, cathodic or both type of corrosion reactions. Unlike the inorganic anodic compounds, they do not have antagonising effect on corrosion when present at lower concentrations in the corroding environment. Another positive feature of the organic inhibitors is that they provide adequate protection at lower concentrations than inorganic compounds. In view of the above-mentioned features of the organic inhibitors the latest trend is to use such compounds in commercial applications.

The organic based compounds no doubt have an edge over the inorganic type of inhibitors but many of them poorly perform in controlling the chloride induced corrosion, especially in alkaline environments such as in concrete. This poor inhibition is due to formation of thin and porous inhibitors’ film on the metal surface allowing chloride to break the protective layer and react with metal surface. To minimize the chloride attack on reinforced steel bars it is essential that the added inhibitor transform into anionic form and adsorb at inner Helmholtz plane of the double layer. This negatively charged layer discourages anionic chloride ions to attack the metal surface.

1,2,3 Benzotriazole (BTAH) and its derivatives are well known inhibitor and widely used for protection of copper and copper-based alloys in different types of environments^11–13^. They are also reported to protect steel in alkaline environment though with poor inhibitive effects^14,15^. Some investigators have reported their corrosion inhibition performance for reinforced steel rebars in concrete environment^16–18^. To achieve good protection, the concentration of BTAH and derivatives need considerably higher concentrations (normally > 1.0%)^14^. Use of high concentrations of these compounds in concrete to achieve adequate protection is not cost effective as well as in many instances unfavourable to other properties of the concrete. In addition to these drawbacks, the efficacy of BTAH based compounds is known to deteriorate after ingress of chloride ions in concrete. To overcome these problems, it was planned to improve the inhibitive performance of BTAH added at low concentration in wet concrete combining it with non-ionic surface-active agent (NIS). The non-ionic surfactants are known to boost properties of additives as well as reduce dosing to get optimum results, employed for different applications including corrosion protection^19,20^. This communication aims to achieve complete protection of rebars embedded in concrete mortars exposed in saline solution for extended period of time with wet/dry treatment by using a synergistic mixture of BTAH and non-ionic surface-active agent as well as impart improved workability of wet concrete, compressive strength, chloride permeability and water absorption.

## Experimental details

### Test material

Thermo—mechanically treated steel rebars of 16 mm diameter used for this study had following chemical composition:

$$ {\text{C}} = 0.{31};\;{\text{Si}} = 0.{22};\;{\text{Mn}} = 0.{86};\;{\text{S}} = 0.0{1};\;{\text{Cr}} = 0.0{1};\;{\text{P}} = 0.0{3};\;{\text{Ni}} = 0.0{3};\;{\text{Cu}} = 0.0{4} $$

All elements’ concentration is given in weight percentage.

The bars had an approximately 2-mm-thick tempered martensite structure rim around their outer diameter, whereas the core structure was pearlite-ferrite. The microstructure of the tested steel is reported in our earlier published paper^21^.

To remove mill scale and rusts from the surface, the rebars were abraded on motorized wheel fitted with 200 grade of emery paper. Before putting the rebars in corrosion cells and mortars their surface was swabbed with ethanol moist tissue paper to ensure removal of any oil or dust.

### Design of corrosion cells

**Figure 1:**
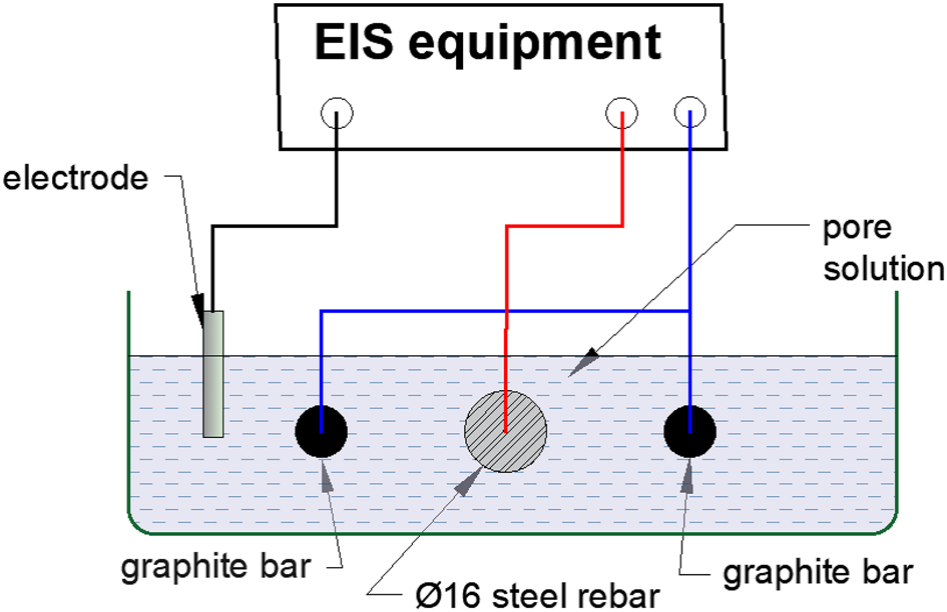
Schematic diagram showing setup of electrochemical cell used to conduct experiments in cement slurry.

To conduct electrochemical tests in cement slurry, de-scaled and abraded rebars of 150 mm in length were fitted in the electrochemical cell shown in Fig. 1. Two graphite rods of 10 mm diameter and of the same length as the bar were also fitted horizontally in the corrosion cell. These rods were short circuited together with copper wire and used as the auxiliary electrode to run the electrochemical experiments. The test rebar was fitted 20 mm besides these rods in the cell, as shown in Fig. 1.

The purpose of fixing of two graphite rods in the corrosion cell was to ensure adequate surface area of the auxiliary electrode. The graphite rods and rebars’ ends emerging from the test cell were blocked with epoxy resin to make the cells leak proof. The appropriate leads of the potentiostat cable were connected to graphite rod and rebar ends that was outside the cell. The saturated calomel electrode was used as the reference electrode. A set of two cells, without and with additives were prepared in plastic boxes of one litre capacity and marked as control, NIS, BTAH and NIS + BTAH. In a separate plastic beaker 750 mL of 3.5% NaCl solution was poured followed by adding 150 gm OPC cement. Thereafter 0.05, 0.10, 0.15, 0.20 and 0.25% (with respect to the weight of cement) of NIS and BTAH were added in different beakers. The pH of the content in all beakers was 12.4 indicating that the additives did not affect the alkalinity. For the optimizing the concentrations of mixture of the two additives the concentrations of the mixture were as follows: NIS: BTAH (%) = 0.10 + 0.10; 0.15 + 0.20; 0.15 + 0.25. This followed a vigorous mixing of the contents of beakers using a mechanical stirrer. In mixed mixture the concentration of NIS was restricted up to 0.15% only as its higher content in concrete mixture made the composition impractical to handle (a higher dosing of NIS greatly enhanced the fluidity of the freshly prepared concrete which remained in fluid state for several days). These thoroughly mixed compositions were then poured in corrosion cells as described above and shown in Fig. 1.

### Design and composition of mortars embedded with rebars

Mortars were cast using ordinary Portland cement conforming to ASTM C150 Type 1, water, and sand (mixed in the proportion of 1:0.5:2, respectively). The sand particle size was within the range of 0.5 mm to 1.00 mm. The casting and curing of the specimens were carried out according to ASTM C192.

**Figure 2:**
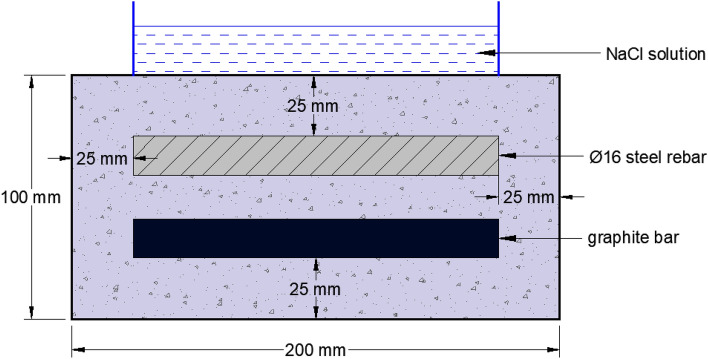
Schematic diagram of the mortar used in this study.

The tests for evaluation of the effect of the additives were performed by embedding the abraded and de-oiled rebars in mortars having the geometry as shown in Fig. 2.

To avoid the effect of the counter electrode geometry on the polarization data, graphite rods with the same rebar diameter (16 mm) were cast in mortar (Fig. 2), and maintained parallel to the steel rebar. This graphite bar was used as a counter electrode during electrochemical studies. To avoid crevice corrosion at the two rebar ends, its 15 mm length at both ends was coated with epoxy and Teflon tape. Insulated copper wire was tightly wrapped on the surface of the rebars and graphite rods before application of tapes and coatings. These wires coming out from the mortars acted as electrical leads to connect the potentiostat. Of the 150 mm length of the rebar samples, only 120 mm was open to exposure in the mortar, so that a 25 mm cover thickness was available for the steel bars from all the casting sides. Three sets of mortars with and without additives were cast abbreviated as follows:

- Control mortars -C;
- mortars blended with NIS:BTAH: : 0.1%: 0.1%
- mortars blended with NIS:BTAH: : 0.15%: 0.20%
- mortars blended with NIS:BTAH: : 0.15%: 0.25%

The mortars were demoulded after 24 h of casting and cured in a humidity chamber maintained at 95% RH and at 25 °C temperature until the age of 28 days.

### Tests procedure

#### Optimization of the composition of the tested additives in cement slurry

To optimize the composition of the mixture (NIS + BTAH) linear polarization resistance (LPR) tests of rebars exposed in cement slurry (composition described in Section “Design of corrosion cells”) were conducted. After 24 h exposure of rebars linear polarization resistance (LPR) tests were performed to determine the optimized composition of the studied inhibitors. This duration of pre-exposure was to ensure the formation of protective passive film on the rebars’ surface^22^. The potentials of the working electrode (± 20 mV) was potentiostatically polarized in anodic and cathodic directions at the scan rate of 0.01 mV/second. The LPR values were computed after drawing tangent lines on plots between current and potential and polarization data determined using the software supplied by the supplier of the instrument.

#### The electrochemical impedance spectroscopy (EIS) and polarization tests

These tests on rebars exposed in cement slurry were conducted on the same samples as those used for LPR (Section “Optimization of the composition of the tested additives in cement slurry”) after leaving the test cells for another 24 h to ensure the stability of the potential of rebars. The tests procedure are detailed in our earlier published papers^23–25^. Before running the polarization tests, electrochemical impedance spectroscopy (EIS) studies were performed by applying a sinusoidal voltage of 10 mV at the open-circuit potential of the working electrode while changing its frequency from 100 kHz to 0.01 Hz. The obtained EIS data were analysed using CMS-300 software (M/S Gamry Instruments). Since EIS is a non-destructive test it is expected that the surface of the tested samples remained unchanged after this experiment. Direct current polarization experiments were conducted on EIS tested samples as per the standard practice of ASTM. All the electrochemical experiments were performed at room temperature (25 °C ± 2 °C).

### Tests on mortars—wet/dry treatment

Wet/dry treatment of mortars accelerates the corrosion rate of embedded rebars. The test procedures to assess the performance of rebars embedded in mortar are described in the authors’ previous papers^21,22^. The rebars embedded mortars were subjected to wet/dry treatments (10 days wet in 5% sodium chloride solution and 20 days dry in laboratory environment). The wet/dry treatments of the rebars embedded mortars were continued up to 43 cycles. After 43 cycles of treatments bleeding spots were noted on the surface of control mortars. At this stage the electrochemical tests were conducted on two sets of all the mortars. The third set of the mortars was broken to examine the surface of embedded rebars. Digital images of the surface of rebars were recorded with camera. Except the rebar embedded in control mortar the other three rebars of inhibitor containing mortars had no sign of visible rust. About 0.5 g of the rust formed on the surface of control rebar was scrapped out and kept in airtight plastic pouches for further investigations. 5-mm length of rebars retrieved from inhibited mortars was cut with diamond cutter machine and kept in airtight plastic pouches for further studies.

### Compressive strength, workability and setting time

The compressive strength of the mortar specimens was determined according to ASTM C109. Mortar cubes were cast adding optimized concentration of the additives as stated in Section “Optimization of the composition of the tested additives in cement slurry”. The cubes were demoulded after 24 h and maintained at 95% relative humidity till the date of testing. Six mortar cubes were tested after 28 days and 90 days. The average results for each additive are presented and discussed in the result section. The workability and setting time of mortars were evaluated by following the ASTM C1437 standard.

### Determination of water absorption through the cast mortars

Effect of addition of the mixed composition of the inhibitors on water absorption was studied as specified in ASTM C 642-06. Cubes of size 100 × 100 × 100 mm were cast and cured as detailed in Section “Design and composition of mortars embedded with rebars”. After 28 days of curing water absorption, test was performed. The values of the percentage retardation efficiencies were calculated by using the procedure as detailed below:

The weights W_**C**_ (weight of cubes after their casting and curing), were recorded up to third decimal point of a gram. There after the cubes were fully immersed in tap water for seven days and then removed and kept in open laboratory atmosphere for 24 h to allow the water on upper surface of cubes to vaporize. The weights of the cubes designated as W_**a**_ (weight of cubes after their immersion in water) were again recorded. The difference in weights (W_a_ − W_C_ = ∆W) provided the weight of water absorbed by the cubes after their immersion in water. Average of water absorbed for three identical control (∆W_control_) and with added inhibitors (∆W_i_) were determined. The percentage retardation efficiency of the inhibitor (%RE) in reducing the permeability of water through the cast cubes were determined using the following equation:

1 $$ \% RE\; = \;\frac{{\Delta {\text{Wcontrol}}\; - \;\Delta {\text{Wi}}}}{{\Delta {\text{Wcontrol}}}} \times 100 $$

### Characterizations of corrosion products

#### X-ray diffraction

The XRD studies were performed using a Siemens D-500 XRD system, Cu-Kα (λ = 1.54 A^0^). Scans were made from the angle of 10° to 90° with scan rate of 3°/min.

#### Scanning *electron* microscopy (SEM) and energy dispersive X-ray analysis (EDXA)

SEM and EDXA analysis of the corrosion products formed on the surface of rebars embedded in mortars were conducted by using Nova NanoSEM-450.

## Experimental results

### Studies on inhibitive effect of additives for corrosion of rebars embedded in chloride added cement slurry

#### Optimization of concentrations of additives for inhibition of rebars exposed in cement slurry using LPR technique

As stated in experimental section of this communication, the optimized concentrations of BTAH and NIS was determined by running linear polarization experiments on rebars exposed in chloride added cement slurry and determined their polarization resistance. The solubility of NIS and BTAH in alkaline pH of cement slurry was not an issue and they freely dissolved in the test environment at all their tested concentrations. The polarization resistance results incorporated in Table 1 show that above a critical concentration of the additives the polarization resistance values remained by and large constant.

**Table 1:** Polarization resistance (R_p_) of rebars exposed in chloride blended cement slurry added with different concentrations of additives and in control environment.

Additives	Concentration, %	R_p_ (KOhm cm^2^)	Inhibition efficiency
Control	0	0.12	–
NIS	0.05	0.315	61.9
	0.10	0.410	70.7
	0.15	1.500	92.0
	0.20	1.48	91.9
	0.25	1.45	91.7
BTAH	0.05	0.13	7.7
	0.10	0.22	45.4
	0.15	0.28	57.1
	0.20	0.30	60.0
	0.25	0.28	57.1
NIS + BTAH	0.10 + 0.10	0.85	85.8
	0.15 + 0.20	9.95	98.8
	0.15 + 0.25	9.85	98.7

Considering that the corrosion rate is inversely proportional to the polarization resistance, percentage inhibition efficiency (%E) of the additives may be calculated by:

2 $$ \% E = \left[ {\frac{{\left( { \frac{1}{c} - \frac{1}{a}} \right)}}{1/c}} \right] \times 100 $$

where ‘c’ and ‘a’ are polarization resistances (R_p_) of the control sample and that of the additives respectively. Data recorded in the above table show that the corrosion inhibition imparted by NIS at all its tested concentration is considerably higher than the corresponding concentrations of BTAH. Further, the optimum concentration for NIS is 0.15% whereas that of BTAH is 0.20%. BTAH alone exhibited very low efficiency (60%). The researchers in the past also reported poor inhibitive effect of this compound (about 70%) for chloride induced corrosion of rebars exposed in simulated pore solution^14^. Polarization resistance of rebars tested under the influence of combination of these two additives mixed at their respective optimized concentrations (NIS = 0.15 and BTAH = 0.20%) is noted to increase appreciably than that tested them individually (Table 1). The inhibition efficiency of combination of optimized concentration of the two additives is 98.8% indicates that the mixed composition of the inhibitor provided almost complete protection to the rebars surface and imparted synergistic protective effect. In light of these findings the optimized compositions of NIS + BTAH (0.15% + 0.20% and 0.15% + 0.25%) were used in all the other studies reported in this communication.

#### Synergistic effect of the mixed additives added in chloride contaminated cement slurry in inhibiting localized corrosion

**Figure 3:**
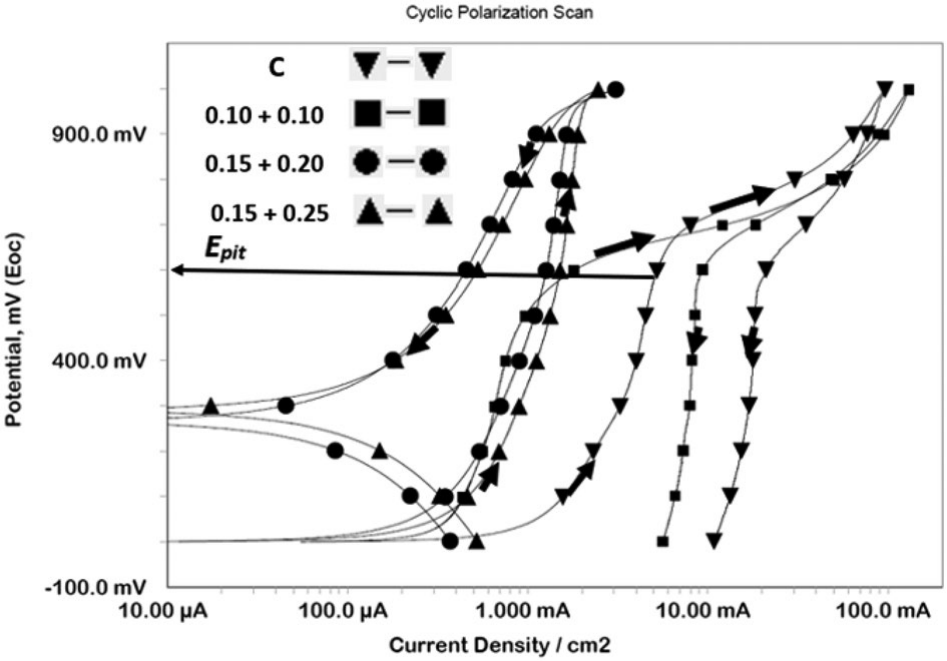
Cyclic polarization curves for rebars immersed in control and mixed inhibitor added chloride blended cement slurry.

Control of localised corrosion of reinforcement caused by chloride ions is of utmost importance for safety and longevity of concrete structures. Out of many techniques to test the efficacy to control pitting corrosion the method of cyclic polarization is simple and yields quick information. To test the effect of mixed additives this method was used for corrosion of rebars immersed in chloride added cement slurry. The forward and back scan rate of potential was kept at 0.167 mV/sec. The cyclic polarization plots for control and mixed inhibitor blended in chloride contaminated cement slurry are shown in Fig. 3. In these plots the anodic potential at which a fast increase in current density during forward scan took place indicated onset of localized attack on the surface of rebars and was taken as pitting potential (E_pit_). It is seen from the figure that the current density suddenly increased at about 600 mv (SCE) (shown by arrow) for control © and low concentration (0.10 + 0.10 concentrations of NIS and BTAH) of additives. In contrast to this the plots for optimized concentration (0.15% + 0.20%) and above it (0.15% + 0.25%) do not exhibit such behaviour. At these concentrations the current density rather remains very low (in μA range) indicating that the presence of optimized concentration of the additives withstood the pitting attack of chloride ions. Another interesting feature noted from these plots is difference between the loop formed in forward and back scan of potentials for control and low concentration of additives and those of ≥ optimized concentrations. In former cases positive loops are formed suggesting that formed pits did not passivate after shifting the potentials in negative directions. Opposite is true in case of later mentioned concentration sets of additives. In these cases, the back scanning of potentials generated lower current than the forward scan of potential and forms negative loops. Such loops lead to the conclusion that any damage caused to the passive film by chloride ions during forward scan was re-passivated during the back scanning of potentials and discouraged chloride ions’ interaction with the steel surface.

#### Synergistic effect of the mixed additives in inhibiting chloride contaminated cement slurry studied by EIS

**Figure 4:**
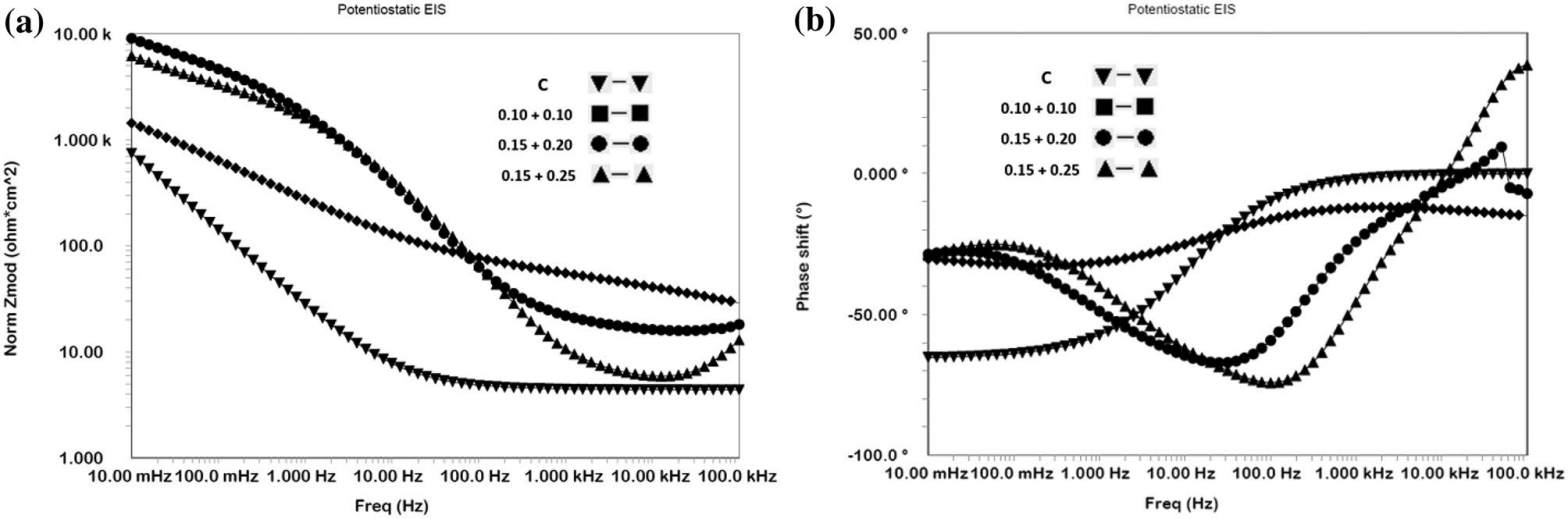
(**a**) Bode frequency—impedance plots for mix composition of NIS + BTAH added in chloride blended cement slurry. (**b**) Bode frequen–y—phase shift plots for mix composition of NIS + BTAH added in chloride blended cement slurry.

The three mixed concentrations of NIS and BTAH tested in chloride added cement slurry were also investigated by using EIS technique. The impedance and phase shift plots in Bodes form are shown in Fig. 4a,b.

The nature of plots for mixed NIS and BTAH tested at different concentrations by and large appear similar except the variations in impedance at the lowest studied frequency. This value of impedance also known as Z_max_ is related to corrosion resistance of a corroding interface. It is seen from the Fig. 4a that Z_max_ for mixed inhibitors is invariably higher than the control sample. Further these values are very close for 0.15 + 0.20% and 0.15 + 0.25% composition of the inhibitor. A similar trend was also noted for performance tests conducted in cement slurry using LPR technique (Table 1). The log frequency-phase shift plots shown in Fig. 4b also exhibit similar characteristics except control sample where the maxima in the curve is shifted in lower frequency region. The two optimized concentrations of the mixed inhibitor exhibit maxima closer to each other and shifted in higher frequency region (approximately 100 Hz).

**Figure 5:**
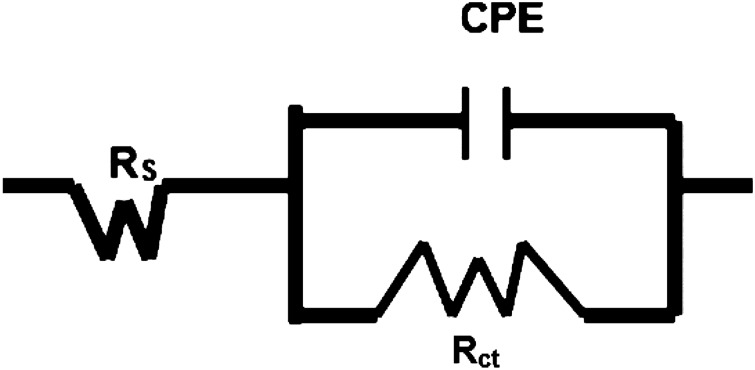
Schematic presentation of electrical components associated with reactions taking place at the rebars surface.

The quantitative data for the protective effect of the added additives were extracted by fitting the data in simulated equivalent electrical circuit. Several permutations and combinations of the elements of the electrical circuit revealed that a circuit comprising of constant phase element (CPE), the series resistance (R_s_) related to the solution ionic resistance, charge transfer resistance (R_ct_), schematically shown in Fig. 5 provided the best fit results with the least error.

In the above schematic diagram CPE denotes –n—homogeneities of the surface^25^ which is expected in the present case due to the formation of corrosion products/passive layer at the steel surface.

The extracted quantitative data from the impedance plots of Fig. 4a,b are recorded in Table 2. The values of the double layer capacitance (Cdl), thickness of the film formed on rebars surface and inhibition efficiencies were computed from the impedance data.

**Table 2:** Electrochemical impedance parameters extracted from impedance plots of Fig. 4a,b using simulated electrical circuit schematically presented in Fig. 5.

Id of samples	Rs (Ω cm^2^)	Rct (KΩ cm^2^)	Y0 (S s^α^/cm^2^) × 10^−6^)	α	Cdi (μFcm^−2^)	t, nm	%E	χ^2^ × 10^−5^
Control	22.46	0.81	481.2	0.56	14.04	2.8	–	1.20
0.10NIS + 0.10 BTAH	13.13	2.65	361.0	0.58	12.70	3.1	70.5	3.41
0.15NIS + 0.20 BTAH	11.80	9.83	212.2	0.58	2.83	14.0	91.7	1.11
0.15NIS + 0.25 BTAH	14.65	9.98	214.2	0.57	2.83	14.0	91.9	2.44

It is noted from the above table that charge transfer resistances (R_ct_) for the exposed rebars in the presence of mixed inhibitors are higher than the control rebar. The R_ct_ values for NIS + BTAH combinations at concentrations of 0.15 + 0.20 and 0.15 + 0.25 are considerably increased in comparison to the control rebar. The charge transfer resistance of the corroding interface is directly related to its corrosion resistance. The values of Y_0_, denoting the admittance of the corroding interface are significantly reduced in case of the mixed inhibitor. The decrease in admittance is considered a diminished electrochemical activity at the interface and is related to the protective nature of the corroding interface. A higher value of Y_0_ on the other hand is attributed to the porous nature of the film through which charge transfer reactions occur. These facts further confirm that the addition of mixed inhibitor in the chloride added cement slurry considerably withstands the chloride induced corrosion of rebars. The term ‘α’ in the above table is a CPE factor and its values range between 0 and 1 depending on the nature of the corroding interface. When the interface behaves as resistor, the value of ‘α’ = 0 and in case of pure capacitor, ‘α’ = 1^26–28^. The values of ‘α’ recorded in Table 2 suggest that both the uninhibited (control) and inhibited interfaces behaved very close to a resistor (‘α’ = 0.56–0.58)) The last column of the table incorporates the values of chi square (χ^2^). The value of chi-square (Χ^2^) is standard deviation recorded for the fitting of experimental data in the model equation and indicative of the validity and acceptability of the fitted data. In the above table the chi-square values are of the order of 10^−5^. It is suggested that the values of chi-square for a good fitting should be below 10^−3^. Macdonald^29,30^, Ren et al.^31^ and Zhao et al.^32^ on the other hand suggest that the chi-square factor is a subjective value and many times it may lead to biased results. They opine that for the reliability of the data the 10^−3^ criteria should not be a deciding factor. Since the fitting of the data using the simulated equivalent circuit of Fig. 5 was good and Kramers–Kronig validity test with chi-square values also ranged between 10^−5^ and 10^−7^, the extracted data recorded in Table 2 are expected to have least error. CPE parameters can be used to estimate the double layer capacitance (C_dl_) as suggested by Bryan Hirschorn et al.^33^. The Cdl values were estimated by using the following equation^33^:

3 $$ {\text{C}}_{{{\text{dl}}}} = {\text{Y}}0^{{{(1}/\alpha )}} \cdot {\text{R}}_{{\text{s}}}^{{\left( {{1} - \alpha } \right)/\alpha }} $$

The data incorporated in Table 2 show that the double layer capacitance values for inhibited interfaces are considerably lower than that recorded for the control interface. These results indicate that the added inhibitors effectively blocked the interface and protected it from the attack of aggressive species (chloride, oxygen moisture etc.) present in the cement slurry.

The accessible area (AA) on the surface of rebars through the pores of protective film formed by the optimized concentration of the inhibitor may be computed from the C_dl_ data of control and inhibited interface by using the following equation^34^:

4 $$ AA = \left( {C_{dlinh} / C_{dlControl} } \right) \times 100 $$

Taking the average value of C_dl_ for the optimized concentration as 2.83 μF cm^−2^ and that of control sample (14.04 μF cm^−2^) the accessible area is 20%. This value is a bit higher than the previously reported data (12%) by Baux et al.^34^ for inhibitive effect of octadecyl amine on steel exposed in alkaline solution. The C_dl_ data can be used to compute the film thickness on the exposed rebars by using the equation:

5 $$ C_{dl} = \varepsilon \varepsilon_{0} / t $$

where ε_0_ = 8.8542 × 10^−14^ F cm^−1^ is dielectric constant of vacuum, ε and t respectively are the permittivity and thickness of the surface film. XRD studies of the surface film indicated that common phase of maghemite was present in all the cases (control as well as inhibited interfaces). In view of this the permittivity of maghemite (4.75) was taken to compute the values of thickness of film formed on the exposed rebars surface. The results recorded in Table 2 reveal that the film thickness on inhibited surface is considerably higher than the control sample. It is to be noted that the thickness of film on metals surfaces greatly varies on measurement techniques, test environment, temperature etc. An exhaustive literature search reveal that the majority of information on film thickness on steel bars pertain to their exposure either in simulated pore solution or in alkaline environments. Thus Ghods et al. using XPS depth profiling technique reported the thickness of 5 nm for the passive film on steel rebars exposed in saturated lime solution^35^. Al-Negheimish et al. employing the same method found the film thickness of 7 nm for carbon-steel bars exposed in simulated pore solution^21^. The results for the film thickness determined by electrochemical methods are a bit lower than those computed by XPS depth profiling. Lv and Li employing Mott–Schottky analysis reported the thickness of 0.1 to 0.9 nm for steel in contact of different pH of simulated concrete pore solution^36^. Similarly, Ismail and Ohtsu using impedance technique found the double layer capacitance (C_dl_) = 12 μF cm^−2^ for steel rebars embedded in concrete. This translated to the film thickness of 1.9 nm^37^. The thicknesses of film formed on steel rebars’ surface during the present study are reasonably in good agreement with the data reported by the above referred researchers.

The % inhibition efficiency of the inhibitor using charge transfer resistance was computed by using the relation:

6 $$ {\text{\% IE}} = \left\{ {\left( {Rctinh - R_{ctcontrol} } \right) / R_{ctinh} } \right\} \times 100 $$

The inhibitor’s efficiency at its optimum concentration recorded in Table 2 exhibits that it imparts the protection of the order of 91%. This value of the efficiency computed from R_ct_ is a bit lower than that evaluated by the linear polarization resistance (LPR) method where efficiency for the same material and environment system is of the order of 98% (Table 1). Such discrepancies in inhibition efficiency by LPR and EIS methods are expected and attributed to changes in interface during the running time of experiments^38^.

### Effect of additive in controlling the corrosion of rebars embedded in mortars

The data generated for corrosion of rebars embedded in mortars are closer to real field conditions. As stated in experimental section of this communication the mortars embedded with rebars were given wet/dry treatments in saline solution. Due to porous nature of mortars chloride ions from the solution migrate and accumulate at the interface of concrete pore solution and rebars. Bleeding at the surface of control mortars appeared after 42nd cycle of w/d treatments. At this stage it was decided to test one set of mortars using different electrochemical techniques and details are described under the following sections.

#### EIS tests

**Figure 6:**
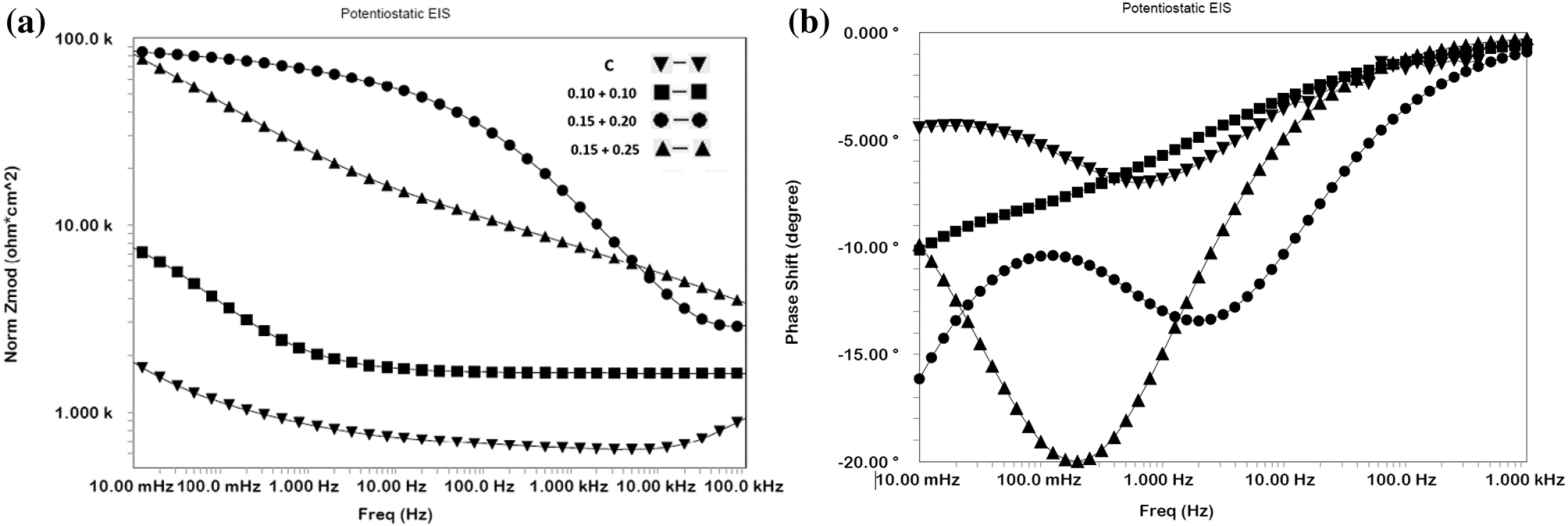
(**a**) Bode log frequency—impedance plots for mix composition of NIS + BTAH added in mortars. (**b**) Bode log frequency—phase shift plots for mix composition of NIS + BTAH added in mortars.

**Figure 7:**
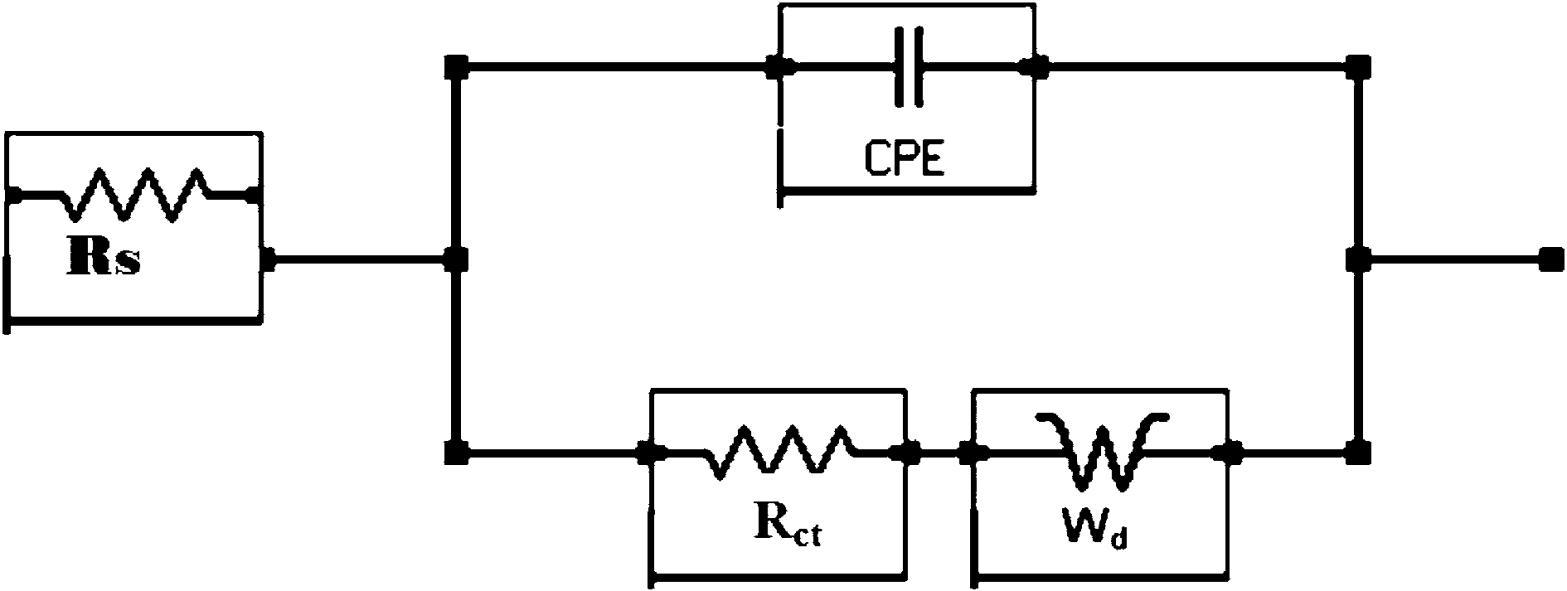
Equivalent electrical circuit of the CPE model incorporating the CPE and Warburg diffusion element.

Since EIS is considered as non-destructive technique the same sets of mortars (control and added with three concentrations of the inhibitor) test were tested using EIS and polarization techniques. The EIS plots in Bode form are presented in Fig. 6a,b. A perusal of the Fig. 6a reveals that the trend of performance of the mixed composition of the additive added in mortars is similar to that noted in cement slurry described in Section “Synergistic effect of the mixed additives in inhibiting chloride contaminated cement slurry studied by EIS”. In this case also the values of Z_max_ for ≥ optimized concentration of additives added mortars are close to each other. Extraction of quantitative data from the above plots was attempted using different simulated electrical circuit models. Unlike the case of rebars immersed in cement slurry where the best fitting of impedance data was achieved using CPE elements in simulated circuit incorporating un-compensated resistance (R_s_), charge transfer resistance (R_ct_) and CPE element, a satisfactory and best fitting of the data in the present condition was recorded after a new component, the Warburg element (W_d_) was incorporated in the circuit presented in Fig. 7. W_d_ is semi-infinite diffusion impedance and relates to mass transport hindrance experienced across the interface of the passive film on metal surface and the test electrolyte. The extracted impedance data for the corroding rebars after 43 cycles of wet/dry treatments are recorded in Table 3. To get best fit the values of ‘α’ for C and lower concentration additive added mortars were in the range as noted for cement slurry experiments (Table 2). The values of charge transfer resistance (R_ct_) for the inhibitor added mortars were significantly higher than the ‘C’ mortar. A phenomenal increase in R_ct_ was noted when optimized concentrations of the additive were added in the mortars (values increased about 20-fold in comparison to control (‘C’) mortar (Table 3). Diminished values of Y_0_ (admittance) are noted for the inhibitor added mortars than the control mortar ‘C’. As stated above (Section “Synergistic effect of the mixed additives in inhibiting chloride contaminated cement slurry studied by EIS”) a lower value of Y_0_ is associated with feeble electrochemical activities at the interface. Both of these parameters of EIS (R_ct_ and Y_0_) strongly support that the optimized concentration of the investigated additive very effectively controlled the deterioration of rebars exposed in mortars.

**Table 3:** Electrochemical Impedance parameters, double layer capacitance, % Inhibition efficiency and thickness of passive film formed on the surface of rebars embedded in mortars determined after 43 cycles of wet/dry treatments of mortars.

Id of samples	Rs (KΩ cm^2^)	Rct (KΩ cm^2^)	Y0 (S·s^α^/cm^2^) × 10^−6^)	Α	Cdi (μFcm^−2^)	t, nm	%E	W_d_, S × s^1/2^/cm^2^ × 10^−6^	(Χ^2^) × 10^−3^
Control	1.61	1.04	42.4	0.63	8.9	4.47	–	1.9	5.8
0.10NIS + 0.10 BTAH	1.22	9.34	33.0	0.58	3.25	12.26	88.8	3.2	2.7
0.15NIS + 0.20 BTAH	1.19	23.25	32.3	0.63	3.13	12.73	95.5	5.4	2.9
0.15NIS + 0.25 BTAH	0.89	19.22	31.7	0.55	1.69	23.57	94.6	4.0	0.25

The values of Cdl, film thickness and inhibition efficiency for mortar embedded rebars were computed using the above Eqs. (3), (5) and (6). The double layer capacitance values for inhibited interface are significantly lower than the control mortar. A comparison of this parameter between the mortar and cement slurry embedded rebars (Tables 2 and 3) shows that the capacitance values are invariably higher in latter condition than the former one. These results suggest that the protection afforded by the studied compositions of the inhibitor in mortars is higher than the cement slurry. This effect is also manifested in results of charge transfer resistance (R_ct_) and inhibition efficiency (%IE). The thickness of the film formed on the surface of mortars embedded rebars is also greater than that recorded for the cement slurry (Tables 2 and 3). This improved performance may be attributed to a longer duration of exposure of rebars in former case (43 months) than the latter situations (48 h).

The component ‘W_d_’ (Warburg diffusion element) incorporated in Table 3 relates to diffusivity of oxidant (D_O_) and reductant (D_R_) as well as oxygen and chloride ions through the corroding interface. This component is related with oxidant, reductant and chloride ions diffusivity and may be presented by the following equation^39,40^:

7 $$ W_{d} = \frac{1}{\sqrt \omega }\left( {1 + j} \right)\left\{ {\frac{RT}{{\sqrt 2 n^{2} F^{2} }}\left[ {\frac{1}{{\sqrt {D_{0} } C_{0} }} + \frac{1}{{\sqrt {D_{R} } C_{R} }}} \right]} \right\} $$

C_O_ and C_R_ in the above equation stand for the bulk concentrations of diffusing species, n is the number of electrons transferred, F is Faraday constant and ω represents radial frequency. As evident from the above equation the value of Warburg diffusion element is inversely related to diffusion coefficient and concentration of oxidizing and reducing components and their decreased values would increase W_d_. The results of ‘W_d_’ incorporated in the above Table 3 with their higher values for the additive added mortars vis-à-vis control mortar suggest that the concentrations of active species (chloride ions, oxygen etc.) across the corroding interface was considerably reduced due to the presence of the mixed inhibitors. These results further confirm the conclusion drawn from the other elements namely R_ct_ and Y_0_.

#### Potentiodynamic polarization

**Figure 8:**
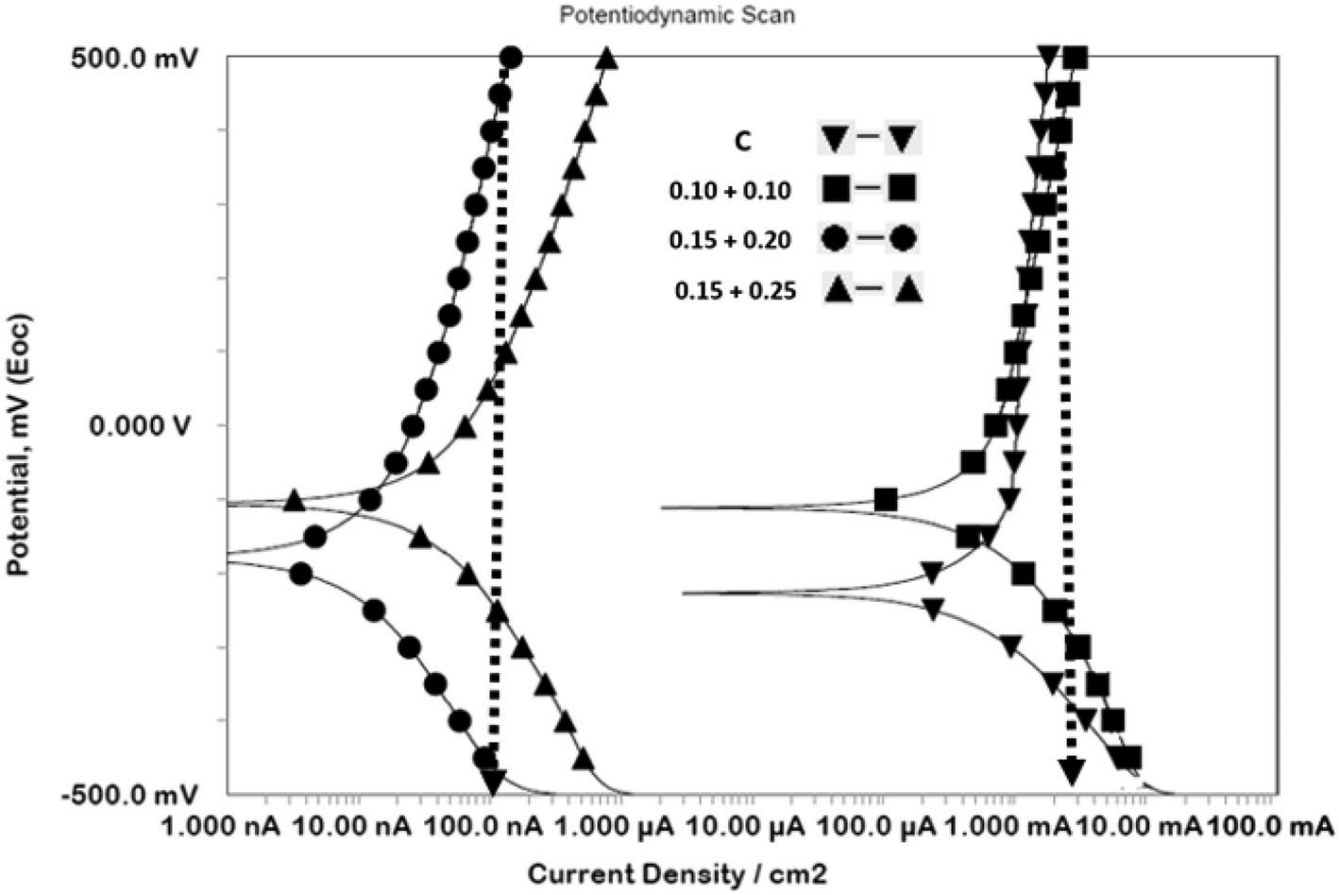
Cathodic and anodic polarization of rebars embedded in control and inhibitor added mortars components of the inhibitors (0.15% NIS + 0.25% BTAH) enhanced the protective properties of the passive film on rebars embedded in mortars by 20,000-fold.

Efficacy of the mixed inhibitor compositions added in mortars were investigated by conducting anodic and cathodic polarization experiments. The polarization plots are shown in Fig. 8. It is seen that the curves for mortars blended with ≥ optimized concentrations of the inhibitors are bodily shifted in lower current density zone. Very little effect is noted on current with rise in potential, for ‘C’ and 0.10 + 0.10 mortars. This current density which is of the order of 2 mA/cm^2^ may be taken as passive dissolution current density. For optimized concentration this value of current density is about 100 nA/cm^2^. These results suggest that addition of an optimized concentration of the two.

#### Cyclic polarization

**Figure 9:**
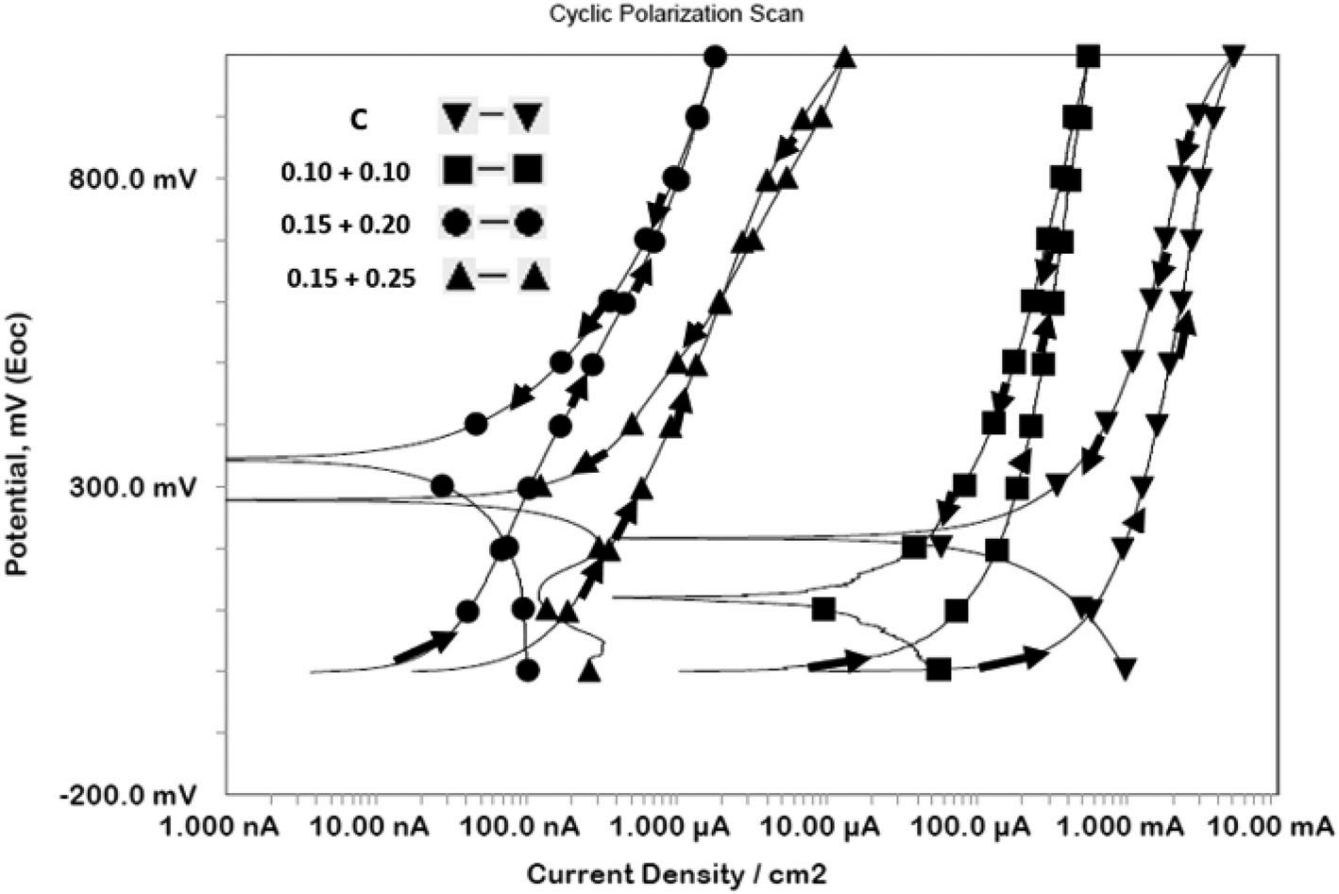
Cyclic polarization curves for rebars embedded in control and mixed inhibitor added mortars after 43 cycles of wet/dry treatments in saline water.

It was important to test whether the mixed composition of inhibitors protected rebars embedded in mortars against chloride induced corrosion. Cyclic polarization tests after 43 cycles of wet/dry treatments on mortars were conducted and plots are presented in Fig. 9. The forward and back scan of potentials are indicated by arrow marks on the curves. It is seen from the curves that in none of the mortars (control as well as inhibitor added) exhibit steep rise in current density up to the potential of 1000 mV.

In all the cases negative loops are noted. The curve for optimized composition mortar (NIS:BTAH: : 0.15 + 0.20 composition) has considerably shifted in lower current density region. These observations suggest that prevailing conditions at the corroding interface either was not aggressive enough to cause pitting attack or applied anodic potential was low for onset of pitting. The findings however suggest that the added mixed composition of the inhibitor strongly inhibited chloride induced corrosion of embedded rebars.

### Visual observations of rebars retrieved from the mortars after 43 cycles of wet/dry treatments

**Figure 10:**
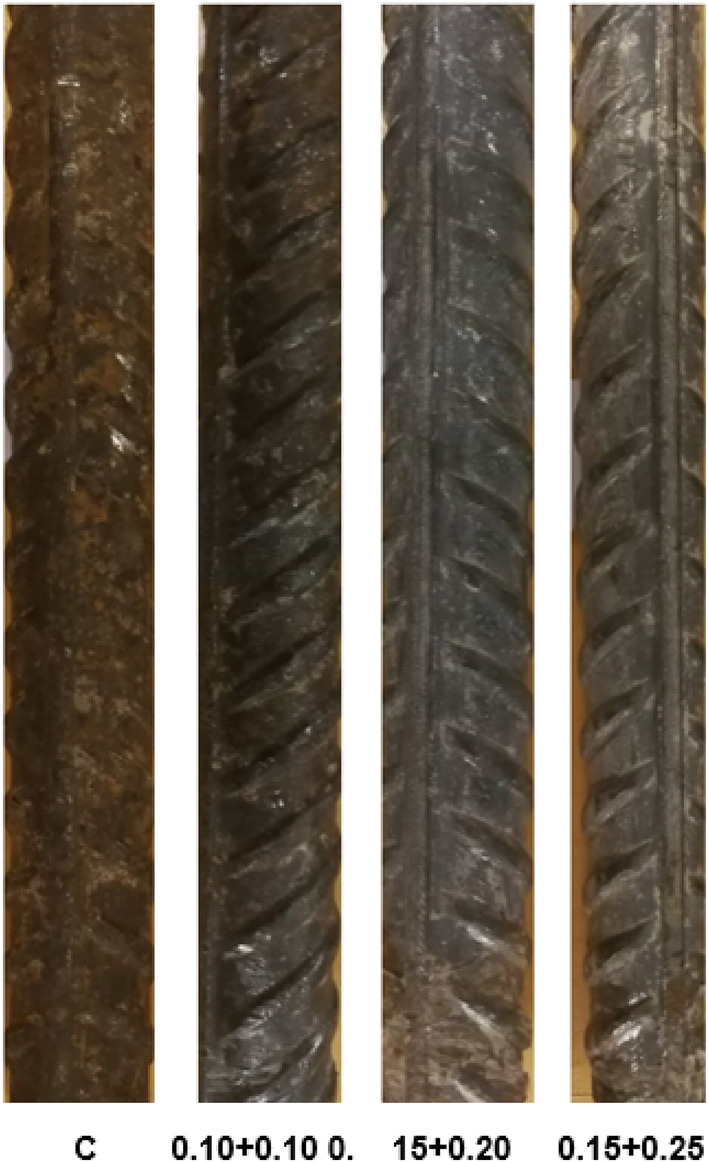
Digital images of mortars embedded rebars retrieved after 43 cycles of wet/dry treatments.

The digital images of rebars retrieved from the mortars after 43 cycles of wet/dry treatments are shown in Fig. 10. The images reveal that rebar from mortar C was severely corroded and whole surface covered with thick brown rust. The rebars retrieved from inhibitor added mortars looked sound. The efficacy of the protection was 0.10 + 0.10 < 0.15 + 0.20 < 0.15 + 0.25.

### Effect of added inhibitor on plasticity of wet concrete and compressive strength of cured mortars

Effect of addition of inhibitors at their different concentrations on plasticity of wet concrete and compressive strength of cured mortars at 28 days and 90 days of curing are recorded in Table 4. It is noted from the table that BTAH adversely affects the plasticity of the wet concrete at w/c of 0.4. At w/c of 0.3 however, it has no effect on flow of wet concrete. PESMO addition in wet concrete on the other hand considerably improves the plasticity at both the studied w/c composition. Very appreciable increase is noted for w/c of 0.30 at all of its studied concentrations. Mixing of BTAH with PESMO adversely affects the plasticity of the wet concrete mixture at both w/c compositions.

**Table 4:** % Flow and compressive strength at 28 days (CS28) and 90 days (CS90) at W/C ratio of 0.40 and 0.30.

Additives	w/c = 0.35			w/c = 0.30		
	%F	CS28	CS90	%F	CS28	CS90
0 (C)	25	73.5	90.3	2	44.5	79.4
0.25BTAH	22	60.4	84.4	2	42.4	76.5
0.50BTAH	21	60.3	76.8	2	40.8	75.9
1.0BTAH	17	57.6	80.5	2	40.3	71.0
0.10% PESMO	48	48.1	61.3	15	62.7	88.3
0.15% PESMO	61	25.0	35.4	21	44.9	86.0
0.25% PESMO	73	8.59	16.4	23	46.7	86.6
0.1PESMO + 0.25BTAH	49	19.2	28.9	15	62.7	88.3
0.15PESMO + 0.25BTAH	49	11.3	16.8	22	44.9	89.0
0.15PESMO + 0.50BTAH	48	9.4	17.7	22	46.7	86.6

The combined mixtures of the two additives namely PESMO and BTAH exhibit considerably greater plasticity than the control samples (about 2wice for w/c = 0.35 and about 7–8 fold for 0.30 water to cement ratio.

### Effect of inhibitors on chloride permeability through the cubes

Rapid chloride permeability tests for control and inhibitors added mortars were conducted as specified in ASTM C1202. Cylindrical plain concrete specimen of approximately 100 mm in diameter and 50 mm in thickness were cast in triplicate using ASTM C150 Type 1 cement, sand and water in the ratio of 1:0.5:2. The test results for average of three samples are shown in Table 5. The cylinders of 28 days age cast with 0.10 + 0.10 inhibitor cracked at 125 min of charging and the test terminated automatically. The identical sets of cylinders at 90 days of age however remained intact at 360 days of charging and also considerably diminished the charge passed through the cylinder (about 35%, Table 5) vis-a-vis control sample. All the other samples withstood the charging of 360 min. Cracking of cylinders added with 0.10 + 0.10 inhibitor composition at 28 days of age probably took place due to incompatibility of lower concentration of the added inhibitor. The best results are recorded for the composition with optimized concentration of inhibitors (0.15 + 0.25 composition). Under this condition the recorded charge for 90 days age sample is 1831 coulombs. According to AASHTO T 277–83, the charge passed through the concrete cylinder in the range of 1000–2000 is low. These results suggest that the mixed combination of the inhibitor in addition to controlling of chloride induced corrosion is also effective in reducing the chloride permeation.

**Table 5:** Chloride permeability through control and inhibitors added cylinders.

ID of cubes	Age of cylinders			
	28 days		90 days	
	Time of test (minutes)	Charge (coulombs)	Time of test (minutes)	Charge (coulombs
Control	360	13,775	360	10,277
0.10 + 0.10	125*	5355	360	6737
0.15 + 0.20	360	11,300	360	3220
0.15 + 0.25	360	11,010	360	1831

*The test was terminated due to high temperature.

### Effect of inhibitors on water absorption through mortars

The role of the mixed compositions of the inhibitor in retarding the permeation of water through mortars was studied using procedure. The results are summarized in Table 6. The data recorded in the table reveal that blending of the inhibitor considerably reduced the water absorption. The blending of the optimized composition of inhibitor was the most effective resulting in reduction of more than 58% water absorption.

**Table 6:** % retardation efficiencies (RE) of the studied inhibitors on permeability of water/moisture through the cured cubes.

ID of cubes	∆W_A_ (gram)	Average ∆W_A_ (gram)	∆W_P_	Average ∆W_P_ (gram)	%RE
Control (C_1_)	11.051	11.049	–	–	–
Control (C_2_)	11.055		–	–	–
Control (C_3_)	11.043		–	–	–
(0.10 + 0.10) mixed composition	–	–	7.275	7.202	34.81
-Do-	–		7.129		
-Do-	–		7.202		
(0.15 + 0.20) mixed composition	–	–	4.832	4.818	56.39
-Do-	–		4.801		
-Do-	–		4.822		
(0.15 + 0.25) mixed composition	–	–	4.583	4.582	58.52
-Do-	–		4.585		
-Do-	–		4.580		

### Morphology of corrosion products and passive film

**Figure 11:**
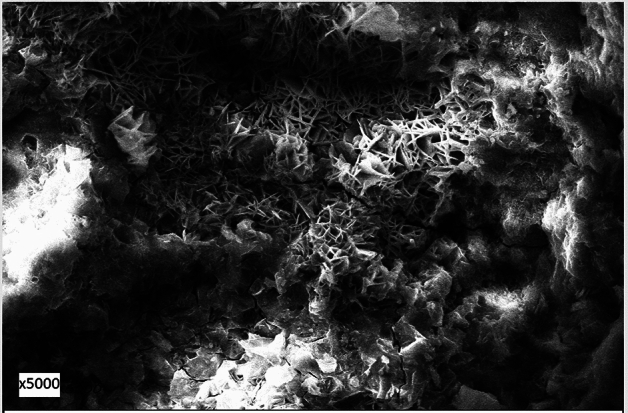
Morphology of corrosion products formed on the surface of rebar embedded in control mortars.

**Figure 12:**
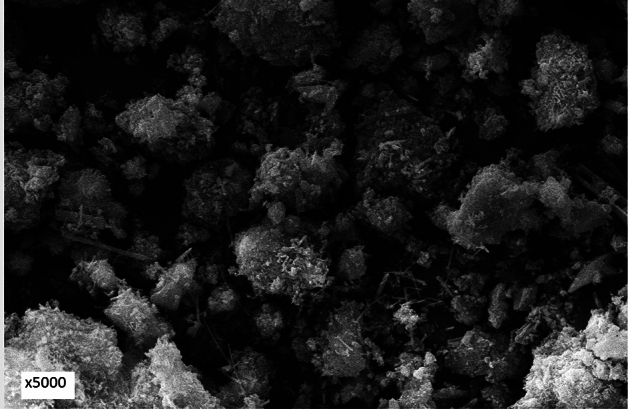
Morphology of corrosion products formed on the surface of rebar embedded in 0.10 + 0.10 inhibited mortars.

**Figure 13:**
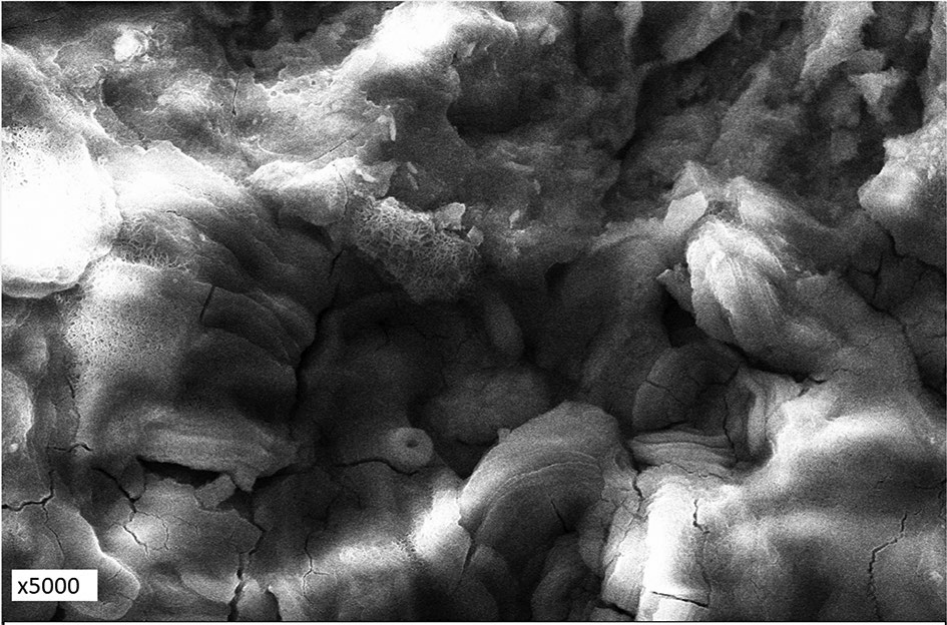
Morphology of corrosion products formed on the surface of rebar embedded in 0.15 + 0.20 inhibited mortars.

**Figure 14:**
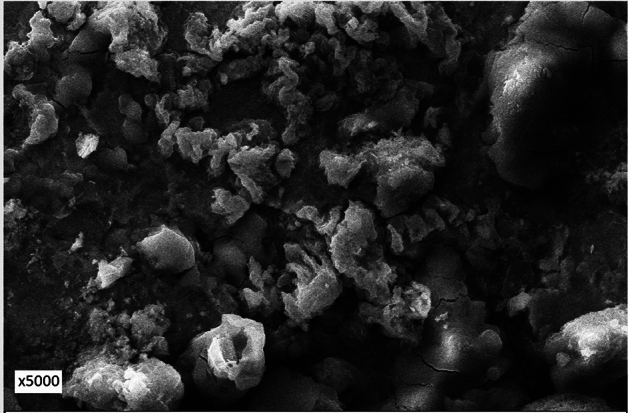
Morphology of corrosion products formed on the surface of rebar embedded in 0.15 + 0.25 inhibited mortars.

Morphology of corrosion products of the surface of rebar embedded in control mortar and passive film formed on the surface of rebars embedded in inhibited mortars were examined by Scanning Electron Microscope. The microphotographs showing the morphologies are presented in Figs. 11, 12, 13, and 14. The corrosion products formed on the surface of control rebar appear quite fluffy, crystalline and porous in nature (Fig. 11).

The surface of inhibited rebars (0.10 + 0.10, 0.15 + 0.20, 0.15 + 0.25) did not exhibit any visible brown rusts. In view of this small pieces of their samples were cut and subjected to SEM examinations. The 0.10 + 0.10 inhibited rebar exhibit comparatively denser and less porous image vis-à-vis control rebar (Fig. 12). The film on 0.15 + 0.20 inhibited rebar is quite compact but cracks in the film are noted at some locations (Fig. 13). In contrast to this the film formed on the surface of 0.15 + 0.25 rebar looks compact with little cracks (Fig. 14). These results corroborate the findings on inhibitive performance of the latter two compositions where a phenomenal improvement in their efficiency was noted (Tables 1, 2, and 3).

### X-ray diffraction of corrosion products

**Figure 15:**
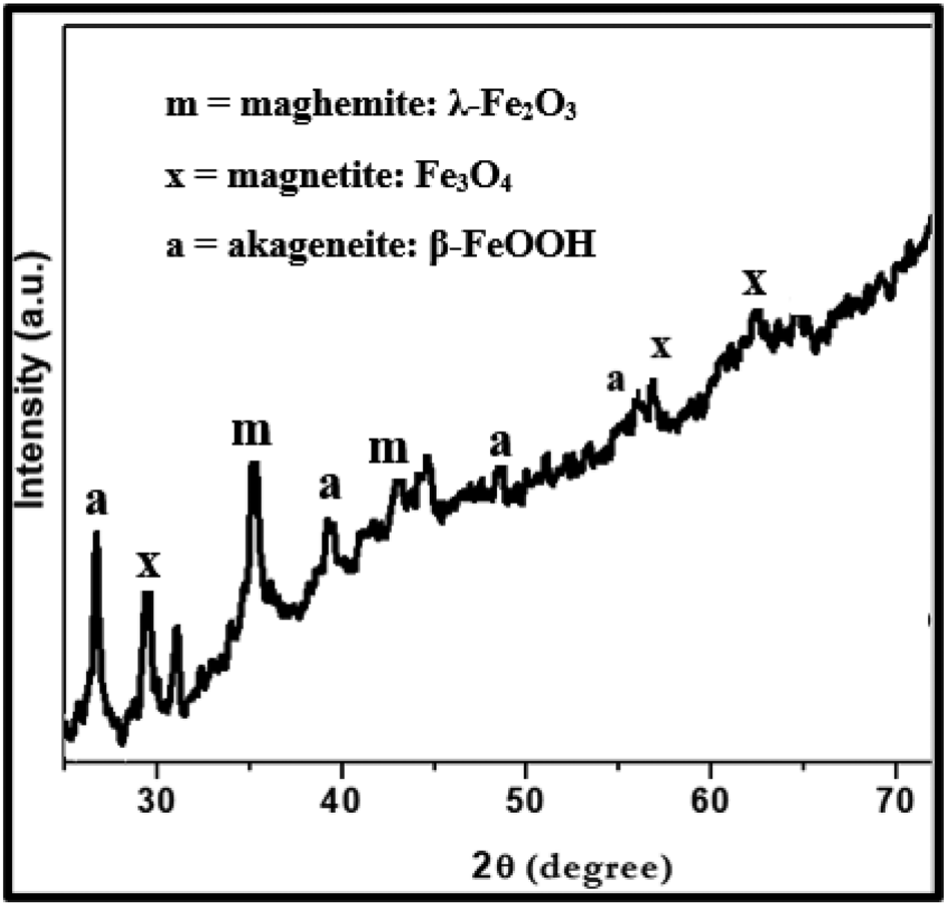
XRD of corrosion products formed on the surface of rebar retrieved from control ‘C’ mortar after 43 cycles of wet/dry treatments.

Corrosion products and film formed on the surface of exposed mortars were analysed by XRD. Since no visible powder was present on the surface of rebars embedded in inhibited mortars a small piece was cut from the rebars and their exposed surfaces were subjected to XRD studies. The diffraction pattern for control sample ‘C’ is presented in Fig. 15.

The analysis of the peaks identified them as maghemite (λ-Fe_2_O_3_), magnetite (Fe_3_O_4_) and akageneite (β-FeOOH). All these peaks are oxides of iron. Out of these peaks the most conspicuous is the presence of akageneite. As shown in subsequent figures, this peak was not traced in XRD spectra of rebars embedded in inhibited mortars. Akageneite phase of iron oxide is formed in corrosion products of iron and steels under specific circumstances. To form this phase of oxide it is necessary that sufficient chloride ions are available at the corroding interface.

**Figure 16:**
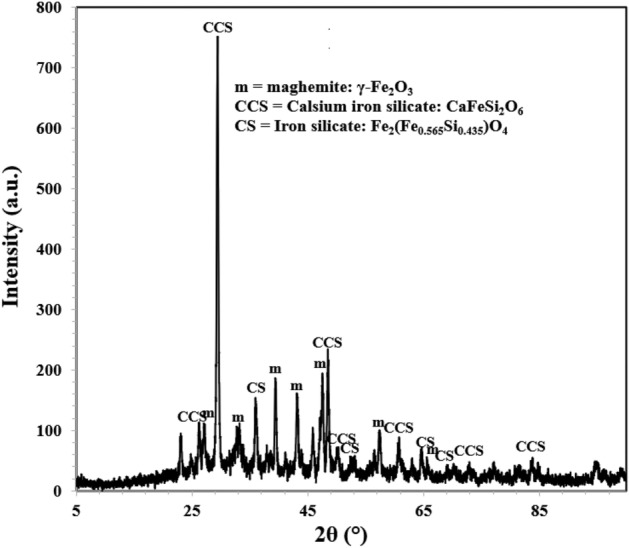
XRD of corrosion products formed on the surface of rebar retrieved from mixed 0.10 + 0.10 inhibitor mortar after 43 cycles of wet/dry treatments.

**Figure 17:**
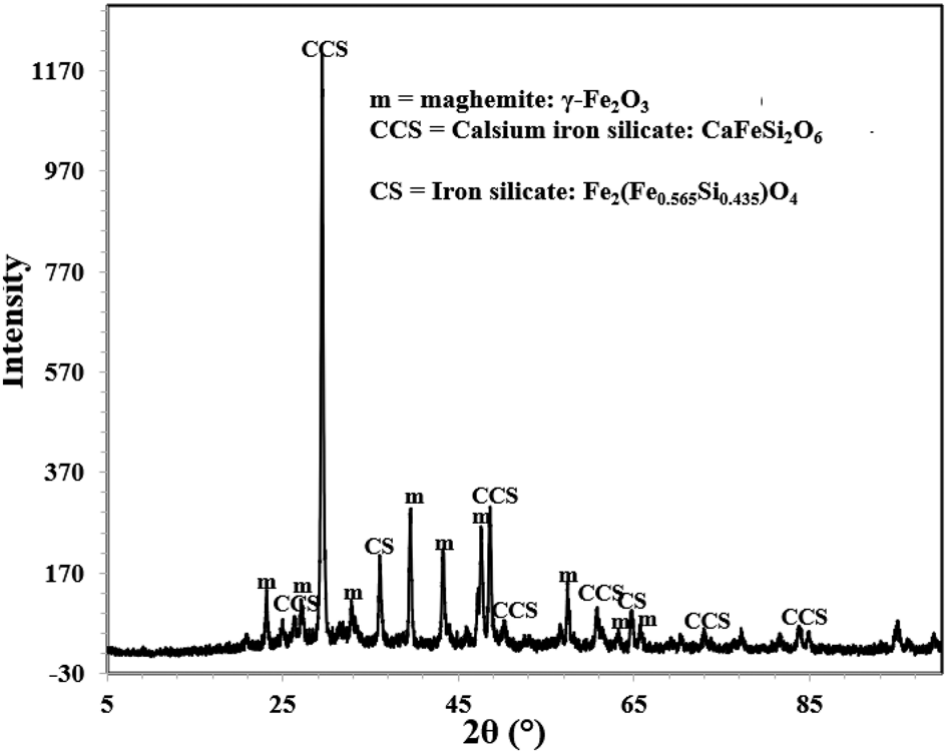
XRD of corrosion products formed on the surface of rebar retrieved from mixed 0.15 + 0.20 inhibitor mortar after 43 cycles of wet/dry treatments.

**Figure 18:**
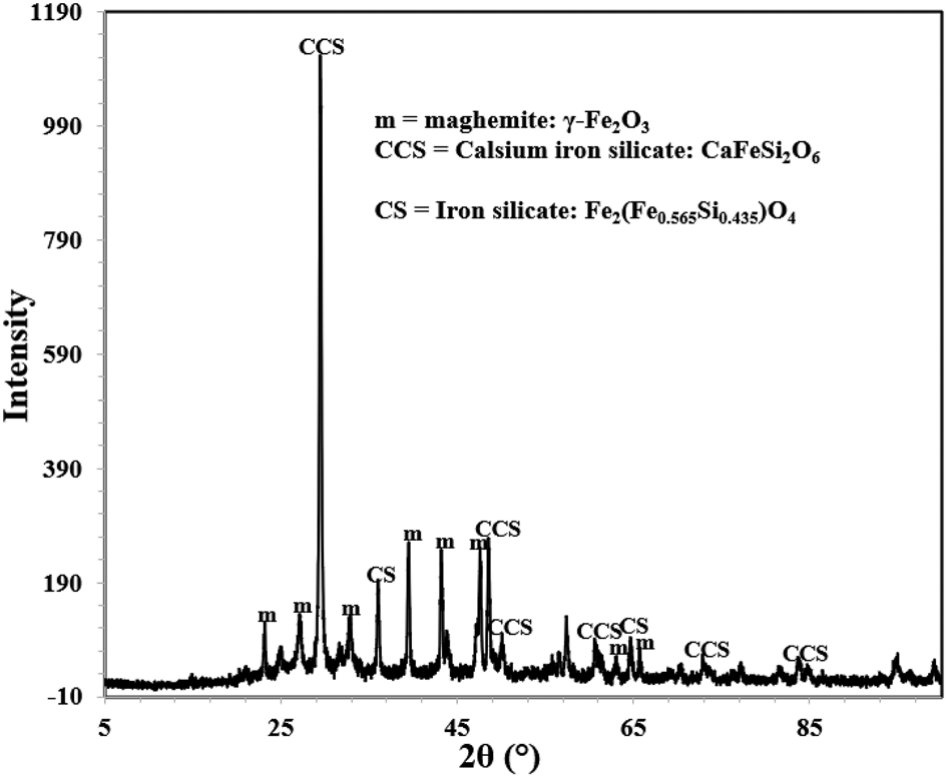
XRD of corrosion products formed on the surface of rebar retrieved from mixed 0.15 + 0.25 inhibitor mortar after 43 cycles of wet/dry treatments.

Identification of multiple peaks of akageneite in corrosion product formed on the surface of rebars embedded in control mortars suggests that chloride from the ponded saline solution had migrated at the rebars surface in sufficient quantity. The XRD spectra of rebars surfaces retrieved from the mortars blended with 0.10 + 0.10, 0.15 + 0.20 and 0.15 + 0.25 compositions of inhibitor however did not yield any peak of akageneite (Figs. 16, 17, and 18). In all the cases identical peaks of maghemite (λ-Fe_2_O_3_), calcium iron silicate (CaFeSi_2_O_6_) and iron silicate (Fe_2_(Fe_0.565_Si_0.435_)O_4_) were detected. Maghemite (λ-Fe_2_O_3_) is a stable phase of rust and passivating type of inhibitors added in concrete facilitate the formation of this phase on steel surface^41^. It appears that the studied composition of the inhibitor facilitated the reaction of silicate of concrete with iron surface forming very adherent passive film on rebars’ surface.

Absence of akaganeite phase on the surface of these rebars suggests that the added inhibitor in concrete helped in formation of densified and defect free passive layer and prevented chloride ions react with steel surface.

**Figure 19:**
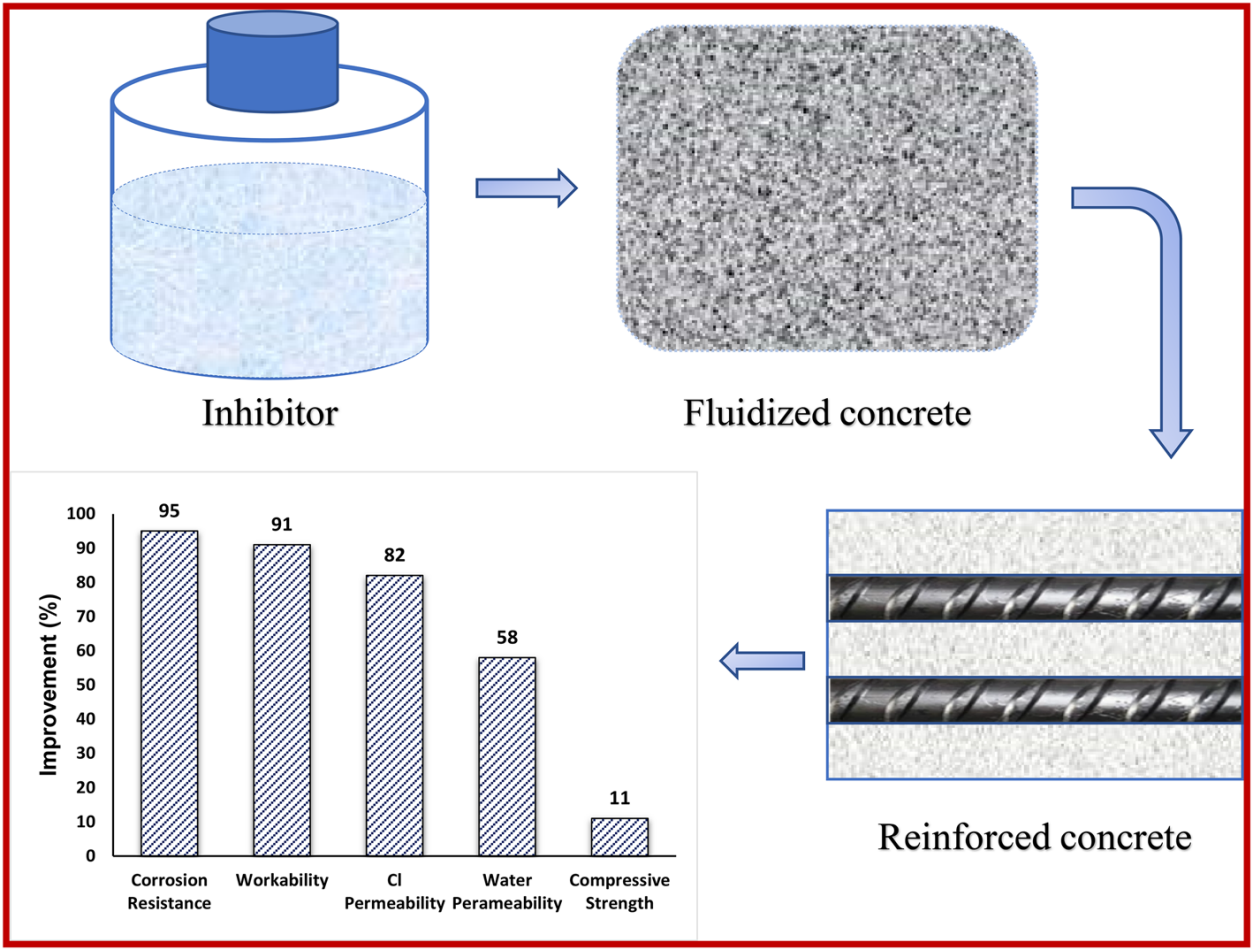
Graphic summary showing the salient improvements after mixing the inhibitor in concrete.

The salient points of the above results may be summarized with the sketch diagram shown in Fig. 19. It may be seen from this figure that mixing of the inhibitor in the fresh concrete increased the workability of the concrete by 91%, corrosion resistance of embedded rebars by 95% and compressive strength of cured mortars by 11%. The chloride and water permeability through the cast and cured mortars decreased by 82 and 58% respectively.

## Discussion

**Figure 20:**
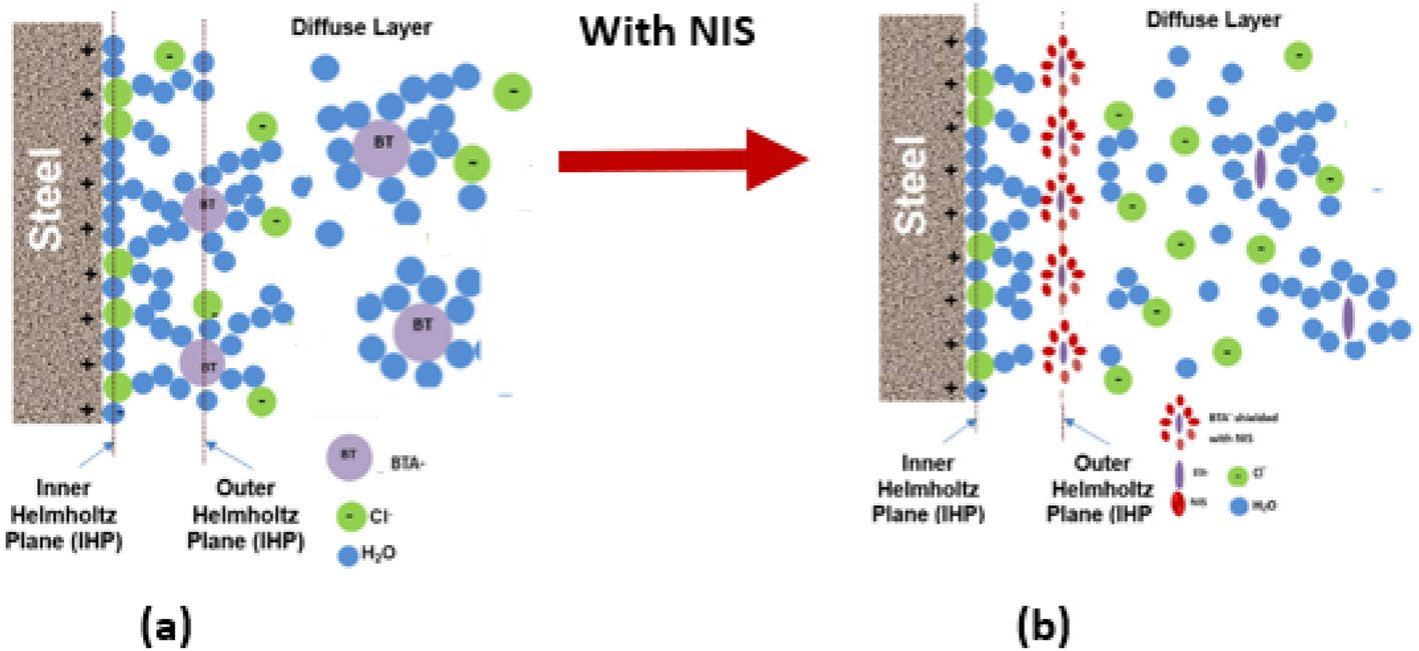
Graphical representation of effect of NIS in modifying the electrical double layer; (**a**) double layer in absence and (**b**) in the presence of NIS.

The results of the present study show that the investigated mixture of corrosion inhibitors very effectively controlled chloride induced corrosion of rebars embedded in mortars. Chloride ions possess very high charge density with great affinity to interact with the metals surface. To minimize the interaction of chloride ions with metals surface it is essential that the metal gets a shield of film with negative charge all over its surface. One component of the mixture of the inhibitor namely benzotriazole (BTAH) is expected to fulfil this requirement. BTAH contains benzene and triazole rings. De-localized p-orbital electron cloud at N atom and on aromatic ring has strong tendency to interact with vacant d-orbital of iron^42,43^. In alkaline solution BTAH ionizes to form anionic specie as shown below^37^:
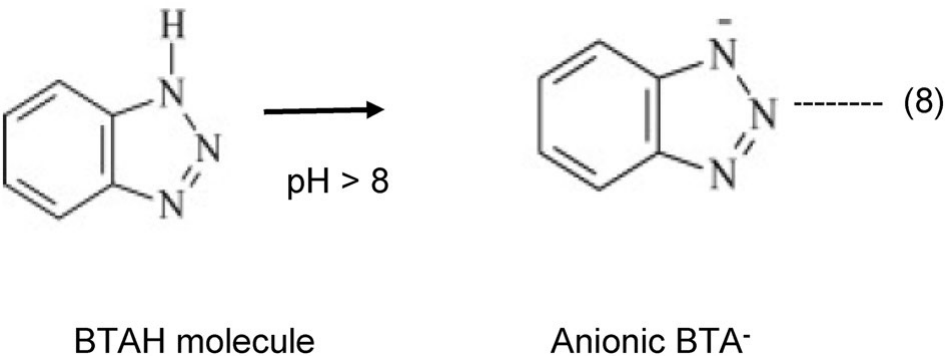


The protective boosting of BTAH by NIS may be explained by considering the changes in electrical double layer after its addition in the test electrolyte (schematically shown in Fig. 20). As seen from the Fig. 20a, in absence of any inhibitor the inner Helmholtz plane (IP) is predominantly adsorbed with water as its concentration at the interface is higher than any dissolved solute^44^ (e.g. pure water contains 55.5 mol dm^−3^). The chloride ions with low hydration energy and strong adsorptive tendency with metals also get interspersed in between^45^. The BTA^−^ anions are unable to attach closely to the surface owing to the presence of chloride and water in IHP and align themselves in close proximity of the IHP forming another layer known as outer Helmholtz plane (OHP). This layer passes through the center of the solvated cations at the distance of their closest approach to the electrode. Only a few BTA^−^ anions are adsorbed in outer Helmholtz plane of the double layer owing to their hydrostatic repulsive effect^46^.

This adsorption partially shields the rebar surface with negative charges, (Fig. 20a). Due to hydrostatic repulsive forces amongst anionic BTA^**−**^, this shielding effect however is limited due to inadequate concentration of the inhibitor at the corroding interface^46^. Very poor inhibition efficiency shown by BTAH alone added at its fairly higher concentration (BTAH showed merely 60% IE at its concentration of 0.25%, Table 1) may be attributed to the effect of this repulsive force. The conjoint effect of NIS and BTAH (0.15 + 0.25) resulted in significant enhancement in the inhibition efficiency (98.8%, Table 1). This synergistic inhibition by the two components of the studied inhibitors may be understood considering the role of the mixed inhibitor in transforming the double layer. Since non-ionic surfactants added in an electrolyte do not ionize in any ionic form, their presence at the corroding interface is not expected to polarize anodic or cathodic corrosion reactions. Their role is simply to minimize the hydrostatic repulsive forces caused due to the ionic species present in vicinity of the interface. The co-adsorption of NIS with other anionic or cationic inhibitors shields them and minimize the repulsion effect^47^. This facilitates the migration of additional ionic inhibitors towards the outer plane of double layer, as shown in Fig. 20b. The increased concentration of BTA^−^ at the corroding interface results in improved inhibition efficiency. This shielding effect by NIS on BTA^−^ appears quite effective especially in controlling the chloride induced corrosion, as evident from the above findings. The accumulation of increased concentration of BTA^−^ anion at the rebars surface discouraged the negatively charged chloride ions to reach at the interface. The absence of akageneite phase in XRD spectra (Figs. 16, 17, and 18) on the surface of rebars embedded in inhibited mortars supports this assertion.

## Conclusion

Mixture of an optimized concentration of polyethoxylated sorbitan mono oleate, a non-ionic surface-active agent and 1,2,3 benzotriazole very effectively control chloride induced corrosion of steel rebars embedded in cement slurry, mortar and concrete. This optimized composition is also effective in retarding the water absorption and chloride diffusion through the mortars. SEM studies show that the protective layer formed on the inhibited rebars had compact film in comparison to the control rebars. The results indicate that the mixed composition of the inhibitor in addition to strengthening the protective seam on rebars surface also favourably modifies the structure of cast and cured concrete. It is suggested that anionic form of BTAH adsorbs on the steel surface. This negatively charged layer of the inhibitor is further strengthened by NIS minimizing hydrostatic repulsive forces of BTA.

## Data Availability

The datasets used and/or analyzed during the present study are available from the corresponding author upon reasonable request.
